# P03-16 Motivating playgrounds: Understanding how school playgrounds support autonomy, competence, and relatedness of tweens

**DOI:** 10.1093/eurpub/ckac095.052

**Published:** 2022-08-29

**Authors:** Thea Toft Amholt, Birgitte Westerskov Dalgas, Jenny Veitch, Nikos Ntoumanis, Jeanette Fich Jespersen, Jasper Schipperijn, Charlotte Pawlowski

**Affiliations:** 1 Department of Sports Science and Clinical Biomechanics, University of Southern Denmark, Odense, Denmark; 2 Institute for Physical Activity and Nutrition (IPAN), School of Exercise and Nutrition Sciences, Deakin University, Melbourne, Australia; 3 KOMPAN Play Institute, KOMPAN A/S, Odense, Denmark

**Keywords:** Tweens, school playgrounds, psychological needs, self-determination theory, physical activity

## Abstract

**Background:**

Physical activity (PA) is an important factor contributing to general health. PA declines rapidly during tween years (9-12 years) when children's social world changes. During tween years, children's self-consciousness develops and their focus on social status, friendships, and appearance increase. It is therefore important to consider how to motivate tween's to be physically active. School playgrounds can contribute substantially to children's PA and are considered key contexts for children to be physically and socially active. Despite the potential of school playgrounds, little is known about how to motivate tweens to use school playgrounds.

Use of motivational theories in health research has increased rapidly in the last 20 years and the Self-Determination Theory has been used to conceptualize and analyze motivation. Using the three basic psychological needs (autonomy, competence, and relatedness) proposed by the Self-Determination Theory, this qualitative study aimed to investigate how school playgrounds can support tweens to enhance their autonomous motivation for PA on school playgrounds.

**Methods:**

We interviewed 56 tweens in focus group go-along interviews in their school playground.

**Results:**

We found that playgrounds could support each of the three basic psychological needs. School playgrounds supported the need for autonomy when the tweens could freely choose from different play equipment pieces. Furthermore, playgrounds should provide enough space to avoid noisy areas and congestion. To support competence, playgrounds should facilitate challenges at different levels, enabling the tweens to practice and experience task mastery. These challenges should contain an element of risk where the tweens can experience a high degree of competence. Playgrounds provide a unique possibility for making and strengthening social bonds. To support relatedness, playgrounds should include areas to hang out and talk with friends of a similar age.

**Conclusion:**

This research highlighted the importance of incorporating tweens' perspectives in playground design to attract and retain them in play and PA in school playgrounds.

